# Unpacking Exosomes: A Therapeutic Frontier for Cardiac Repair

**DOI:** 10.1007/s11886-025-02225-8

**Published:** 2025-03-20

**Authors:** Elena McMullan, Darukeshwara Joladarashi, Raj Kishore

**Affiliations:** 1 Aging and Cardiovascular Discovery Center, Lewis Katz School of Medicine, Temple University, Philadelphia, PA 19140 USA; 2 Department of Cardiovascular Sciences, Lewis Katz School of Medicine, Temple University, Philadelphia, PA 19140 USA

**Keywords:** Exosomes, Heart failure, Cardiac fibrosis, Hydrogel, Microneedle/Cardiac patch

## Abstract

**Purpose of Review:**

The rising global prevalence of cardiovascular disease is driving the need for innovative biotherapeutics. Recently, exosomes-extracellular vesicles involved in paracrine signaling have shown promise in aiding heart repair associated with cardiovascular conditions. Their therapeutic potential encompasses several beneficial mechanisms, including anti-fibrosis, anti-inflammation, pro-angiogenesis, anti-oxidation, and anti-apoptosis, all contributing to improved cardiac function. This review provides a comprehensive overview of exosomes and highlights the latest research on their effectiveness in addressing current challenges in regenerative cardiac medicine.

**Recent Findings:**

Current approaches revolve around elucidating and enhancing how different cell types, cargo, and delivery methods impact healing in a pathological cardiovascular environment.

**Summary:**

The emerging field of therapeutic exosome research is promising for cardiac regeneration due to the beneficial effects of exosomal cargo. The expansion of mechanistic knowledge and the optimization of techniques are required before standard clinical application.

## Introduction

Cardiovascular disease (CVD) is the leading cause of death globally, affecting over 620,000,000 people [1–3]. In 2021, 20,500,000 deaths resulted from CVD, 9,440,000 of those attributed to ischemic heart disease (IHD) [3]. IHD is characterized by arterial plaque buildup, which reduces blood flow to the heart due to the narrowing of the arteries. Insufficient blood flow starves the muscle tissue of oxygen. The decrease in blood and oxygen leads to myocardial infarction (MI) and subsequent necrosis, or cell death. Fibrosis is when the dead cells are replaced with scar tissue, contributing to left ventricular remodeling [4]. Heart failure advances, as fibrotic tissue is inefficient at muscle contraction. With the rising trend of CVD, the necessity of developing novel, efficient cardiac repair methods become increasingly imperative to decrease the serious burden CVD poses for the healthcare system globally.

## Stem Cell Treatment Inadequacy

The use of stem cells is a therapeutic approach to cardiac repair that supports tissue regeneration via differentiation into the desired cell type. The most extensively studied stem cell varieties are mesenchymal stem cells (MSCs) and induced pluripotent stem cell-derived cardiomyocytes (iPSC-CMs), with MSCs exhibiting paracrine effects and immune privilege [5] and iPSC-CMs augment the pre-existing cardiomyocytes through electrical coupling [6] leading to improved cardiac function post-myocardial infarction. Even though stem cell therapy offers improvement to counteract the effects of cardiovascular disease, it still presents certain drawbacks. Homing, which refers to successful cell delivery and engraftment to the target area, is shown to be weak in stem cells. The cells are at risk of veering off-course to an undesired organ, or for dying post-injection. There is a documented low cell survival percentage in the hostile injured heart microenvironment. Factors contributing to the harsh ischemic environment include the production of reactive oxygen species (ROS), hyperinflammatory responses triggered by proinflammatory cytokines, and apoptosis from oxygen starvation [6]. To counteract the low survival rate, the patient would require multiple doses. This accumulates risk when delivered intramyocardially or intracoronary due to the invasive nature of these injections [7].

Immune rejection is a risk associated with allogeneic stem cell transplantation, because of major histocompatibility complex (MHC) antigen incompatibility between the patient and donor cells [8]. The solution to MHC mispairing is the use of immunosuppressants, which also puts the patient in danger of opportunistic fatal infections. Stem cells are easily induced to differentiate and expand in vitro; however, they are vulnerable to senescence [9], which is the permanent aged phenotype that prevents future cell division and growth. This is an issue for the clinical application of stem cell-based therapies, which requires a significant number of cells, typically between 1 × 10^8 and 1 × 10^9 [10], for the highest efficacy and to counteract the low implanted cell survival rate. Teratoma formation is a safety concern attributed to iPSCs [8]. The formation can be due to residual undifferentiated pluripotent cells with tumorigenic genetic mutations that would not come to fruition until post-transplantation. Avoiding this side effect would require terminal differentiation of iPSCs, careful monitoring and specific tumor-targeting drugs [10]. Despite the self-renewable capabilities of stem cells and the decades of work dedicated to the exploration of their efficacy, exosomes have been shown to have advantages over their stem cell predecessors, bypassing many of the challenges impeding stem cell therapy. The cell-free approach of exosomes has the potential to become the preferred method of therapy in post-MI treatment, focusing on signal delivery rather than cell delivery.

## Exosome Treatment

Exosomes are influential in intercellular signaling, released by all cell types in health and sickness. These nano-sized particles, 30–150 nm in size [11], were once believed to be the mechanism by which cells shed their unwanted contents[12]. Since their discovery in the 1980s, their pivotal role in cellular communication continues to be elucidated. They are an attractive candidate for therapeutics for many reasons, including their low immunogenic properties, high biocompatibility in patients, and stability attributed to the lipid bilayer encapsulating the cargo [13]. Due to their inability to self-replicate, exosome therapy does not have the side effect of potential teratoma formation, as seen in stem cell therapy [14] Their cargo can also be bioengineered for increased specificity, efficacy, and even drug delivery [15].

## Exosome Cargo

Exosomes contain thousands of bioactive molecules, such as proteins, lipids, and nucleic acids, notably DNA and regulatory RNAs such as messenger RNA (mRNA), small non-coding RNA (sncRNA), and microRNA (miRNA) [16]. These are all protected within the confines of their phospholipid lipid bilayer, which also express different types of cell surface proteins [17]. They reflect the contents and physiological state of their parent cell at the time of excretion, also making them helpful biomarkers in assessing disease [18].

## Exosome Biogenesis and Expulsion

The formation of exosomes begins with the inward membrane budding and early endosome formation. In this step, membrane proteins and pieces of cytosol become internalized and taken up by early endosomes. In the endosomal compartment, intraluminal vesicles (ILVs) are generated again by inward membrane budding of the endosomal membrane, in this instance [18]. ILVs serve the purpose of encapsulating bioactive cargo, and their formation marks the maturation of the early endosome into a multivesicular body (MVB). From this stage, MVBs either fuse with lysosomes or the cellular plasma membrane. Lysosome-fused MVBs are degraded, whereas plasma membrane-fused MVBs release their ILVs as exosomes into the extracellular space [19, 20]. The latter method requires regulation by Rab GTPases and SNARE complexes, which contribute to specificity. Rab proteins are a specific type of GTPase, which act as molecular switches between states with bound GTP (guanosine triphosphate), the active state, and bound GDP (guanosine diphosphate), the inactive state. MVBs are brought to the plasma membrane, and activated Rab proteins are involved in docking, trafficking, and SNARE complex activation and recruitment. Active and assembled SNARE complexes work to fuse the two membranes and allow for exocytosis [18, 21]. Once secreted, exosomes are free to transmit signals by binding to receptors on nearby cells for uptake.

## Exosome Uptake

Arrival, docking, and internalization of the shipment of exosome cargo to the recipient port are first determined by interactions between exosome surface ligands and target cell receptors. Membrane proteins on the exosomes, such as tetraspanins, integrins, and major histocompatibility complex (MHC) molecules, bind to receptors of recipient cells and aid trafficking and homing to specific organs. Tetraspanin ligands like CD63, CD81, CD82, and CD9 help characterize exosomes and are involved in cell recognition and binding. Integrins promote homing target specificity and adhesion [22]. MHCs specifically bind and activate T cell receptors (TCRs), influencing immune regulation and communication. Exosome contents are incorporated into recipient cells by either membrane fusion or endocytosis [19]. The membrane of the exosome can fuse with the cellular plasma membrane, leading to cargo release into the cytosol. Natural endocytic mechanisms, including exosome membrane fusion with the endosome membrane and evasion of degradation via lysosome, also facilitate the process of bringing exosome contents into the recipient cell’s cytosol.

## Sources of Exosomes

**Fig. 1:**
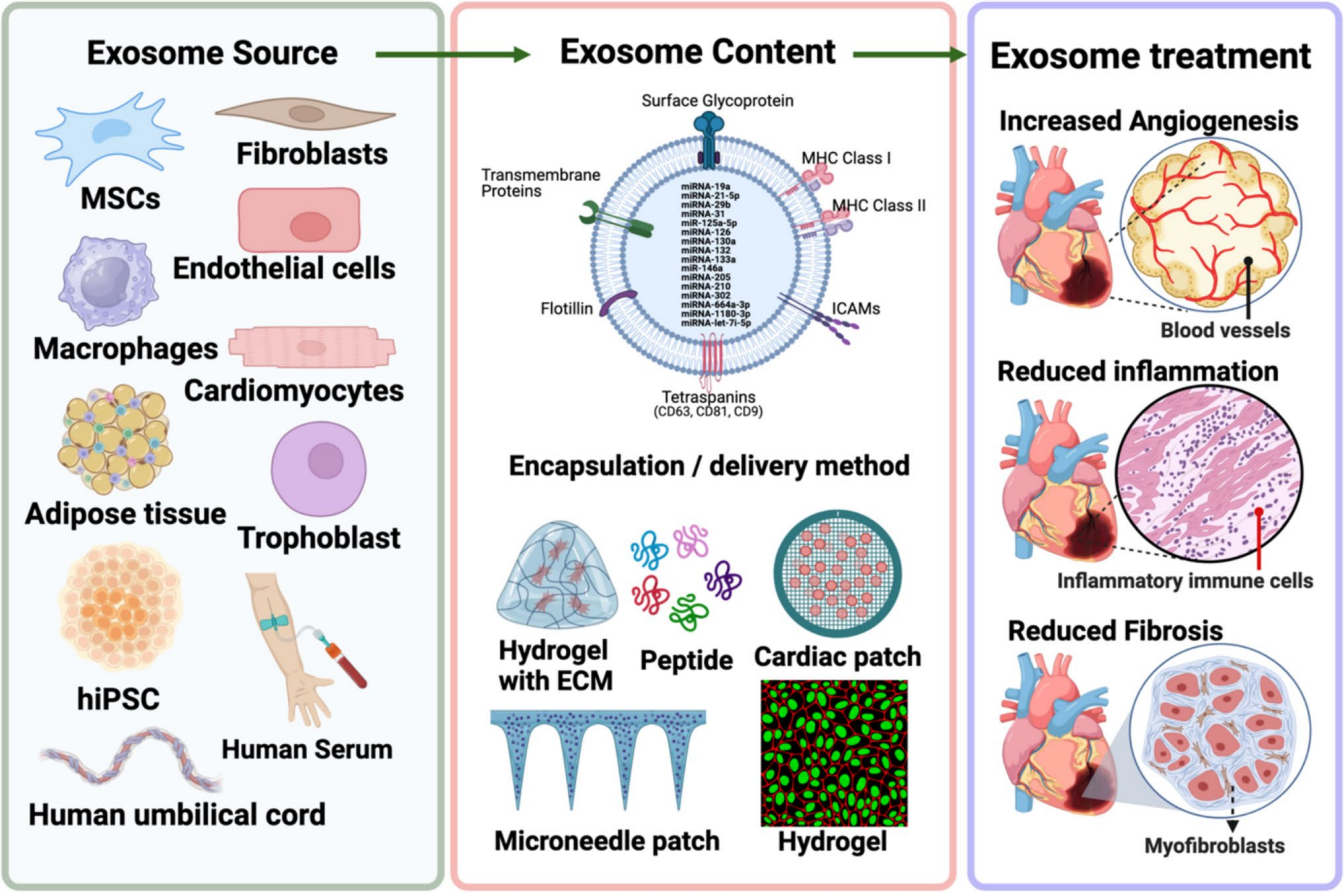
Exosomes for cardiac repair and function. Exosomes isolated from different sources carry and deliver miRNAs and other biomolecules to the damaged heart tissue, consequently promoting cardioprotective effects. Created in BioRender. Gonzalez, C. (2025) https://BioRender.com/b81x338

A plethora of source options for exosome isolation exist since all known cell types produce these vesicles. Exosomes isolated from stem cells are a popular choice, but so are macrophages, human serum, and various types of cardiac cells (Fig. 1). The preferred source of stem-cell-derived exosomes is mesenchymal stromal cells (MSC) that come from bone marrow [23, 24], human umbilical cord [25], or neonatal [26]. Stem cells could also come from human induced pluripotent stem cells (hiPSC) [27], lung spheroid cells [28], adipose stem cells [29], and trophoblasts [30]. Trophoblast-derived exosomes loaded into a controlled-release injectable hyaluronic acid hydrogel were utilized in MI treatment for mice, showing improvement in cardiac function and recovery as characterized by the induction of anti-fibrotic, angiogenic, anti-remodeling and anti-inflammatory effects [30]. M2 macrophages are also a source of exosome therapeutic contenders [31]. Exosomes with pro-inflammatory M1 macrophage parent cells were shown to inhibit cardiomyocyte proliferation [32], whereas anti-inflammatory M2-derived exosomes encouraged proliferation [33]. The exosomes from several kinds of cardiac cells have been used in cardiovascular therapy, including cardiomyocytes, cardiac fibroblasts [34], endothelial cells, cardiosphere-derived cells (CDC) [35], cardiac progenitor cells (CPC) [36] and hiPSC-derived induced cardiomyocytes [27].

## Mechanism

Exosomes are involved in cardio-protection in cardiovascular disease (CVD), promoting cardiac repair and regeneration stimulated by their intercellular messaging capabilities [35]. They act as a package delivery system to the damaged area, bringing molecules that signal activation of angiogenesis, cardiomyocyte survival and proliferation, anti-inflammation, and anti-fibrosis. The​summary of available studies is listed in Table 1.

**Table 1:** List of exosome sources, cargo, and targets of the exosomes in the regulation of cardiac repair

Effect	Exosome Source	Related Exosomal Cargo	Mechanism	Reference
Pro-angiogenic	Adipose-derived mesenchymal stem cell (ADSC)	miRNA-31 miRNA-126 miRNA-130a miRNA-132	Enhance expression of VEGF, EGF, FGF	[29]
	ADSC	miRNA-126	Increase PI3K/Akt signaling	[39]
	M2 macrophage	miRNA-132-3p	Downregulate THBS1 mRNA expression	[33]
	ADSC	miRNA-205	Increase HIF-1 and VEGF expression Promote endothelial cell proliferation	[40]
Anti-apoptotic	Bone marrow mesenchymal stem cell (BM-MSC)	miRNA-let-7i-5p	Upregulate Bcl-2 protein expression Downregulate Bax protein expression	[42]
	Cardiac fibroblast	miR-133a	Downregulate ELAVL1 protein expression	[34]
	Mesenchymal stem cell (MSC)	miR-19a	Inhibit PTEN Activate Akt and ERK pathways	[41]
	MSC	miR-210	Decrease ROS production Stimulate PI3K/Akt pathway	[41]
	Human endometrial MSC	EGF	Activate PI3K/Akt pathway	[44]
	MSC	miR-21-5p	Repress YAP1 Inhibit Hippo pathway	[45]
	BM-MSC	miR-302	Inhibit Hippo pathway	[46]
Anti-inflammatory	ADSC	miR-146a	Inhibit TRAF-6 and IRAK-1 Decrease neutrophil recruitment	[39]
	MSC	miR-125a-5p	Inhibit TRAF6/IRF5 pathway Increase M2 macrophage polarization Decrease M1 macrophage polarization	[49]
Anti-fibrotic	Human umbilical cord MSC	miR-29b	Downregulate Col-I/III and MMP-2/9 mRNA	[53]
	MSC	miR-1180-3p	Inhibit ETS1 signaling	[54]
	Human serum	miR-664a-3p	Inhibit TGF-β/SMAD4 signaling pathway	[55]
	BM-MSC	curcumin	Inhibit TGF-β/SMAD2/3 signaling pathway	[56]

## Exosomes and Angiogenesis

Angiogenesis is essential to cardiovascular therapy to restore blood flow to hearts impacted by ischemia using pre-existing vasculature to generate new blood vessels [37, 38]. This brings about renewed access to nutrients and oxygen carried in the blood. Promotion of angiogenesis can be attributed to multiple microRNAs including mi-R31, miR-126, miR-130a, miR-132 [29], miR-126 [39], miR-132-3p, miR-221-3p, and miR-222-3p [33]. Adipose-derived stem cell (ADSC) exosomes were loaded onto an oxygen-releasing nanofibrous bi-layered cardiac patch, which allowed for a sustained release of oxygen and exosomes. The output showed an increase in angiogenesis and cell survival with a decrease in oxidative stress caused by oxygen deficiency post-MI. In this study by Shiekh et al., showed that, miRNAs miR-31, miR-126, miR-130a and miR-132 contained in ADSC exosomes enhanced the expression of multiple pro-angiogenic growth factors including vascular endothelial, epidermal, and fibroblast [29] Shafei et al. demonstrated the regenerative effectiveness of ADSC exosomes loaded with miR-126 and miR-146a. MiR-126 promotes angiogenesis by enhancing PI3K/Akt signaling [39]. Proangiogenic effects of miR-132-3p in M2 macrophage-derived exosomes were explored by Guo et al. [33]. They demonstrated miR-132-3p, transported to endothelial cells after MI, downregulated THBS1 mRNA expression, encouraging angiogenesis [33]. Experiments by Wang et al. described the promotion of angiogenesis by miR-205 in ADSC exosomes after intravenous injection. Their results showed the promotion of endothelial cell proliferation and increased HIF-1 and VEGF expression [40].

## Exosomes and Apoptosis/Cardioprotection

Cells in a hypoxic environment, like the ischemic myocardium, will overproduce reactive oxygen species (ROS) that create conditions of oxidative stress when not neutralized by antioxidants [41]. In oxidative stress, cellular damage and apoptosis are prone to occur, worsening the vulnerable heart and emphasizing the importance of controlling ROS presence, homeostasis, and cardiomyocyte protection via antioxidation. Bone marrow mesenchymal stem cell (BM-MSC) exosomes expressing miRNA-let-7i-5p, injected into three points immediately following surgical induction of myocardial infarction, protected against cardiomyocyte apoptosis by protein regulation, specifically upregulating Bcl-2 and downregulating Bax protein expression [42]. A study performed by Liu et al. showed cardiac fibroblast-derived exosomes in hypoxic conditions expressed higher levels of miR-133a, which, when delivered to cardiomyocytes repressed inflammation-induced pyroptosis by targeting ELAVL1, a pro-inflammatory RNA binding protein, for downregulation [34]. Liu et al. presented the roles of MSC-derived exosomal miR-19a and miR-210 in apoptosis inhibition after myocardial infarction [34]. MiR-19a injected into the post-MI myocardium reduces damage to the heart by inhibiting target proteins, like phosphatase and tensin homolog (PTEN), and activating the Akt and ERK pathways [43]. MiR-210 is upregulated by transcription factor HIF-1 in hypoxic conditions to decrease ROS production and stimulate the PI3K/Akt pathway [41]. Exosomes derived from human endometrial MSCs loaded into a conductive hydrogel made from poly-pyrrole-chitosan exhibited properties of anti-apoptosis. Yan et al. conveyed that these exosomes contain EGF (epidermal growth factor) proteins that activate the PI3K/Akt pathway [44]. MSC exosomes containing miR-21-5p have been shown to play a role in cardiomyocyte protection by inhibition of the transcriptional coactivator of the apoptosis-associated Hippo pathway, YAP1 (Yes-associated protein 1) [45]. Gu et al. programmed exosomes from bone mesenchymal stem cells to possess a cardiomyocyte specific peptide for target accuracy and loaded the exosomes with miR-302. This microRNA is known to support cardiomyocyte survival and proliferation, also by inhibiting the Hippo pathway [46].

## Exosomes and Inflammation

Inflammation is the body’s natural first response to remove dead cells following myocardial infarction using leukocyte infiltration. Excessive inflammation can lead to heart failure when left unresolved [47]. When exosomes derived from BM-MSCs treated with TNF-α were injected into 5 points around the infarct area, they exhibited cardioprotective effects, reduced infarct size, and promotion of anti-inflammation [23]. Wang et al. observed enhanced M2 macrophage polarization and an increase in the anti-inflammatory cytokine IL-10, in addition to suppressed M1 macrophage polarization and a decrease in the pro-inflammatory cytokine IL-1β [23]. Zhu et al. demonstrated that MSC exosomes injected intrapericardially resolved inflammation and aided in cardiac repair. This is due to the exosomes stimulating the Foxo3 pathway in MHC-II^+^ antigen-presenting cells, in turn supporting regulatory T cells [48]. In combination with pro-angiogenic miR-126, Shafei et al. examined the anti-inflammatory role of miR-146a, which inhibits TRAF-6 and IRAK-1, leading to a decrease in neutrophil recruitment [39]. Following treatment of MSCs with Nicorandil, a cardioprotective drug for heart disease patients, Gong et al. isolated exosomes that were utilized following acute myocardial infarction. They found increased M2 macrophage polarization, stimulated by miR-125a-5p-mediated inhibition of the TRAF6/IRF5 pathway [49].

## Exosomes and Fibrosis

Cardiac fibrosis occurs after myocardial infarction, marked by excessive extracellular matrix and cardiac fibroblast proliferation[50]. The scar tissue that forms is inefficient at muscle contraction, presenting a need for amelioration of fibrosis to increase cardiac competence and reduce risk of complete heart failure [51]. In experiments by Kang et al., they determined that conjugation of human serum exosomes with fibroblast activation protein (FAP) and miR-29b (this miRNA is downregulated in a post-MI environment) showed improved cardiac function and reduced fibrosis. These exosomes also exhibited higher target selectivity because of the natural FAP overexpression in injured cardiac tissue [52]. Yuan et al. isolated exosomes from human umbilical cord MSCs and delivered them in a microneedle patch. Cardiac fibroblasts took up these exosomes and upregulated expression of miR-29b, inhibiting fibrosis by downregulating mRNA in scar-tissue-forming proteins like collagen type I and III (Col-I/III) and matrix metalloproteinases 2 and 9 (MMP-2, MMP-9) [53]. Delivery of miR-1180-3p to cardiac fibroblasts plays a role in inhibiting ETS1 signaling, therefore relieving fibrosis caused by TGF-β−1, as shown by Li et al. [54]. Wang et al. explored cardiac fibrosis inhibition by miR-664a-3p in exosomes isolated from young (18–25 years old) male human serum intramyocardially injected post-MI. The results of the study showed inhibition of fibrosis because of the miR-664a-3p binding to SMAD4 RNA, inhibiting the fibrotic TGF-β/SMAD4 signaling pathway [55]. Wang et al. evaluated the use of extracellular matrix hydrogel with BMSC-exosomes containing curcumin in fibrosis prevention. Curcumin is a compound found naturally in turmeric and works through inhibition of the TGF-β/SMAD2/3 signaling pathway [56].

## Current Issues

Although exosome therapy is a promising field, there are still complications that prevent their widespread use in clinical settings. Some of the major challenges that need to be addressed include targeting, retention, and standardization of isolation. Exosomal cargo release to cells for signaling purposes is high and effective, but only if they can survive long enough in sufficient quantities in the desired area to confer therapeutic benefit. Phagocytosis by macrophages contributes to the limited exosome retention, presenting a need for camouflage proteins in certain exosome membranes [57]. Target specificity is another difficulty pertaining to injection and inhalation methods of exosome administration. While these methods may be safer, patches and hydrogels loaded with exosomes directly installed or injected on the heart provide elevated homing efficacy. Additionally, the lack of official standardization of exosome purification and isolation methods is a limitation. It is crucial for therapeutic exosome samples to be pure (free from other types of extracellular vesicles or molecules) [58]. Technique uniformity and optimization should be a focus moving forward towards the goal of successful therapy.

## Exosome Therapy

The field of exosome therapy requires successful delivery methods in order to provide patients with the full regenerative effects that exosomes have the potential to convey. Injection, whether intravenous or intramyocardial, was the earliest standard, but new methods are being developed today to increase effectiveness. Patches successfully retain the exosomes and allow them to affect the desired area, but they require invasive procedures to be implemented. There is a greater emphasis on minimally or non-invasive strategies such as hydrogel sprays or inhalable exosomes. In murine studies, common methods of injections are intravenous (usually through the tail vein), intraperitoneal, subcutaneous, or local (intramyocardial) [59]. To experience the full effects of treatment, multiple injections are typically needed because of poor retention, homing, and the short half-life of exosomes in organs. Cardiac patches can be advantageous because they allow for extended release, addressing the retention issue and eliminating the need for repeated injections. Unfortunately, their installation is typically invasive [29]. Exosomes isolated from a human umbilical cord mesenchymal stem cell (HUC-MSC) source were loaded into a fibrin patch, mimicking an extracellular matrix as well as allowing for a sustained release of exosomal cargo once attached to the infarcted heart. Wang et al. discovered improved exosome retention in the heart post-MI, inhibition of fibrosis and improved cardiac function [25]. The non-invasive method of inhalation for exosome delivery post-MI was explored by Li, et al., which they call stem cell-derived exosome nebulization therapy, or “SCENT”. In this study, exosomes were isolated from lung spheroid stem cells and administered through a nebulizer, resulting in improved left ventricular function, reduction in fibrotic tissue, and promotion of cardiomyocyte proliferation, without side effects on other organs after treatment in mouse and swine models [28]. Conductive hydrogels enhance exosome retention and utilization. In a less intrusive manner than patches, hydrogels can be injected onto the ischemic myocardium [60]. Recently, a spray made from thrombin and MSC exosomes in a fibrinogen solution, termed “EXOS”, was deposited in vitro through small incisions to mice suffering from MI. The spray formed a gel once on the heart, serving to preserve the stability and contents of the exosomes and counteract the issue of specified organ targeting [61]. The components of hydrogels vary, but the method of deposition is typically consistent. Hydrogels are currently the most attractive option for their properties of biodegradable controlled release, minimally invasive insertion and homing efficacy of exosomes.

## Conclusions

Recently, exosomes have emerged as the future of cardiovascular repair because of their role in paracrine communication, and current research shows evidence of successful cardiac improvement after cardiovascular disease-induced damage. These extracellular vesicles circumvent many obstacles presented by other methods of treatment, such as stem cell therapy. Exosomes are formed and secreted by nearly all cell types and contain many proteins, lipids and nucleic acids that are used for signal transduction. They deliver molecules to target cells that stimulate mechanisms promoting angiogenesis and counteracting apoptosis, excessive inflammation, and fibrosis. Advancing the field of this cell-free approach to therapy is imperative for furthering the treatment of pathological conditions of the heart, reducing the global burden of cardiovascular disease.

## Key References

- Li J, Sun S, Zhu D, Mei X, Lyu Y, Huang K, et al. Inhalable Stem Cell Exosomes Promote Heart Repair after Myocardial Infarction. Circulation. 2024;150:710–23.
  - This article presents the first study on the inhalation delivery of exosomes for treating heart injuries, showcasing their potential to enter circulation, to be directed by the ischemic heart, and to improve cardiac functions in proof-of-concept studies involving rodents and swine.
- Wang W, Li Y, Zhang C, Zhou H, Li C, Cheng R, et al. Small Extracellular Vesicles from Young Healthy Human Plasma Inhibit Cardiac Fibrosis After Myocardial Infarction via miR-664a-3p Targeting SMAD4. Int J Nanomedicine. 2025;20:557–79.
  - This article revealed that small extracellular vesicles from young, healthy human plasma can directly bind to the 3′-untranslated region of SMAD4 mRNA via miR-664a-3p, thereby inhibiting the TGF-β/SMAD4 signaling pathway, which protects the heart from fibrosis and improves cardiac function. Given the ease of obtaining plasma-derived exosomes, this study presents a promising therapeutic strategy for heart failure, with the potential for rapid clinical translation in the near future.

## Data Availability

No datasets were generated or analysed during the current study.
